# Assessment of Risk of Behavioral Problems in Children Below Five Years in Relation to Screen Time: A Cross-Sectional Study

**DOI:** 10.7759/cureus.72459

**Published:** 2024-10-27

**Authors:** Manisha Ghosh Roy, Amit Agrawal, Rajesh Patil, Jyotsna Shrivastava

**Affiliations:** 1 Pediatrics, Gandhi Medical College, Bhopal, Bhopal, IND

**Keywords:** behavioral problems, children below five years, ppsc, prevalence rate, screen time

## Abstract

Background: Excessive screen time is associated with developmental (mainly socio-cognitive and language) and behavioral problems more in children below five years than in older children and adolescents. The present study was conducted to determine the prevalence and pattern of exposure to excessive screen time in children below five years of age. It was also aimed at determining the prevalence of the risk of behavioral problems in these children in relation to their average daily screen time.

Methodology: This study was conducted as an observational descriptive study on children attending OPD in the Department of Pediatrics of a tertiary care medical college in central India over the study period of 12 months. Parents were enquired about their socio-demographic details, the daily average screen time of their child, details regarding screen time and exposures, associated factors, sleeping and eating habits, average time spent by parents with their children, and also the behavioral issues according to the preschool pediatric symptom checklist (PPSC). All these questions were asked of the parents and duly filled out by a single doctor. The IAP (Indian Academy of Pediatrics) screen time recommendation was used as a cut-off value for excessive screen time definition, which states screen time recommendations for children below two years and two to five years old.

Results: The daily average screen time greater than one hour (exceeding the recommended guidelines) was found in 57.7% of children. Twenty-six percent of children were exposed to screen time before their first birthday. Among the study participants, 37.7% (n=95) of children were found to be at risk of behavioral problems as per PPSC score, with a mean PPSC score of 6.7±3.3 in these children. The daily average screen time of one to three hours was found in 39.3% of children, three to five hours in 13% of children, and more than five hours in 5.3% of children. The PPCS score was found to be significantly higher in children with higher average screen time, children using multiple devices as compared to single devices, children who preferred electronic devices or devices with toys as preferred sources of entertainment, children becoming restless when devices are not given, children having feeding and sleep issues, and children who spend less time with parents (p<0.05). Multivariate analysis revealed higher odds of behavioral problems in children having more screen time (1-3 hours OR: 1.536; 95% CI: 1.03-7.237; p=0.035, 3-5 hours OR: 2.022; 95% CI: 1.477-8.570; p=0.029, and >5 hours OR: 9.793; 95% CI: 2.065-46.447; p=0.004), children using multiple devices as compared to single devices (OR: 2.43; 95% CI: 1.194-4.95; p=0.014), and children whose parents spend less time with them (<3 hours OR:1.311; 95% CI: 1.02-2.77; p=0.012).

Conclusions: The present study found that almost all children below five years are exposed to digital devices, with the majority of children exceeding the recommended screen time guidelines, and they mostly use it for entertainment purposes. During this period, exposure of children to screens is significantly associated with the risk of behavioral problems. It was observed that excessive screen time, less time spent by parents with their children, and use of multiple devices are significant predictors of increasing risk of behavioral problems in children below five years.

## Introduction

Screen-based devices are now an integral part of our everyday lives. It has been seen that over the past 20 years, the daily duration of screen time for children has increased while the age at first exposure has decreased [1]. Increased access to digital devices, such as smartphones, tablets, etc., has contributed to a rise in average screen time exposure, especially in children [2,3]. Screen time is the time spent watching any screen-based device like television, computers, gaming devices, mobile phones, and tablets [4]. Certain features of screen-based media can help children better understand media content, including the use of common social characters, simple tasks, or the use of audio and visuals to direct children to important information and learning [5].

Various guidelines have been recommended by various governing bodies to restrict the use of digital media and avoid screen time in young children. The World Health Organization (WHO) recommends the avoidance of screentime in one-year-old children, and it should be limited to less than one hour per day in children between two and five years of age [4]. According to IAP guidelines, children below two years of age should not be exposed to any type of screen except for occasional video calls with relatives, and screen time for children between the ages of two and five years should not exceed one hour. Early exposure to screen time in children impacts a child’s behavior as it tends to form a habit that tracks into adulthood [6].

The prevalence of screen time has been documented to range between 10% and 93.7% in high-income countries, and that in low- and middle-income countries (LMICs) varies from 21% to 98%. It was noted that the group that spends the most time in front of the screen in in-home care is children between the ages of three and five years [7]. 

Just as screen-based media can help in the cognitive and social development of a child, excessive screen time can hamper the growth and development of a child. Excessive screen time has various ill effects on health and overall well-being. Excessive screen time is associated with adverse effects on not only physical health (poor sleep quality, increased risk of noncommunicable diseases in the future, impaired vision, and decreased bone density) but also psychological effects as well as psychoneurological effects [8]. Excessive screen time is associated in a dose-response relationship with behavioral and conduct problems, developmental delays, speech problems, learning disabilities, autism spectrum disorder (ASD), and attention deficit hyperactivity disorder (ADHD). Too much screen time is more associated with developmental and behavioral problems in preschoolers than in children and adolescents. It can be concluded that children who spend too much time in front of screens, especially boys, are at increased risk of developing developmental and behavioral problems [9].

Most of the studies on screen time and behavioral problems in children are carried out in the Western population [4,5,8,9]. There is a dearth of studies evaluating the same in Indian children, especially in preschool children. We believe similar findings can be seen in Indian preschool children, and hence, there is a need for screening of behavioral problems in these children. The present study was conducted to determine the prevalence and pattern of exposure to excessive screen time in children below five years. We also aimed to determine the risk of behavioral problems in these children in relation to their average daily screen time. A study hypothesis was developed that there will be an increased risk of behavioral problems in children with increased screen time.

## Materials and methods

This observational cross-sectional study was conducted on children eighteen months to five years old attending pediatric OPD of a tertiary care medical college in Central India over a 12-month study period from January 2023 to December 2023.

Inclusion criteria

All the children of consenting parents attending the study center during this time period were included.

Exclusion criteria

Children with any known behavioral problems, troubled parents, single parent, parents having known behavioral and psychiatric problems, children with known chronic diseases of any system, and hospitalized children were excluded from the study.

Sample size

The sample size was estimated using the formula:

n= z2pq/d2

where n = sample size; z = 1.96 at 95% confidence interval; p = 77% = 0.77 as per preschool pediatric symptom checklist (PPSC) scale [10]; q = 1 - p = 0.23; d = absolute error = 5% that is 0.05. Based on the above formula, n = 272 cases. Thus, a total of 300 cases were enrolled in the study.

Process

After obtaining ethical clearance from the Institute's ethical committee, all the children fulfilling the inclusion criteria were enrolled and written consent was obtained from the parents or guardians. Detailed socio demographic history was obtained and entered in the questionnaire. Parents of these children were enquired about the daily average screen time of their child, details regarding screen time and exposures, associated factors, sleeping and eating habits, average time spent by parents with their children and also the behavioral issues according to the PPSC. Average daily screen time was the time in approximate hours a child spends watching screen in a day. All these questions were asked to the parents and duly filled up by a single doctor. The IAP (Indian Academy of Pediatrics) screen time recommendation was used as a cut off value for excessive screen time which states screen time recommendations for children below two years and two to five years old. Using the PPSC checklist, a score was assigned for each question ranging from 0 to 2. A score of “0” for each “Not at All” response, a “1” for each “Somewhat” response, and a “2” for each “Very Much” response was assigned as the sum was noted. A total score of 9 or greater was considered as "at risk" of behavioral problems.

Preschool pediatric symptom checklist

The PPSC is an 18-item questionnaire intended to be used in primary care settings to assess children aged 18 months to 5 years old for social and emotional issues. The PPSC also has the advantages of being brief, easy to understand, free of cost, and simple to score. The PPSC's accuracy and dependability are on par with other measures, despite its short duration, with a sensitivity of 93% and specificity of 89% [11].

The PPSC was validated in the local language, and a pilot study was also run to confirm the parents' understanding of the checklist.

Follow-up advised

Subsequently, children who were screened as “at risk” of behavioral problems (PPSC score ≥ 9) were sent to our designated pediatric psychologist for detailed evaluation, diagnosis, and treatment of any behavioral problem if detected and also for frequent follow-ups in our OPD.

Statistical analysis

Data were compiled using MS Excel (Microsoft® Corp., Redmond, WA) and analyzed using IBM SPSS Software version 20 (IBM Corp., Armonk, NY). Categorical data were expressed as frequency and proportions, whereas numerical data was expressed as mean and standard deviation. Data were analyzed using the chi-square test (for categorical data). Multivariate analysis was done to determine the risk factor of behavioral problems. A P-value of less than 0.05 was considered statistically significant.

## Results

A total of 300 cases were enrolled in the study with a mean age of 44.14 ± 13.36 months (range: 18 to 60 months). The majority of children belonged to 49 to 60 months of age (36.7%), and about 56.7% of cases were males. About 46% of the fathers were either graduates or postgraduates, whereas 22% of the mothers were graduates. About 99.7% of fathers and 18.7% of mothers were employed. About 34.7% and 34.3% of children belonged to the lower middle and upper lower classes, respectively. The majority of parents, i.e., 62% of parents, spent more than three hours per day with their child, and in the absence of parents, 96.7% of children were attended to by other family members (Table 1).

**Table 1:** Distribution according to sociodemographic variables

Baseline variables		Frequency (n=300)	Percentage
Age (months)	18–24	42	14.0
	25–36	54	18.0
	37–48	94	31.3
	49–60	110	36.7
Sex	Male	170	56.7
	Female	130	43.3
Father’s education	Illiterate	13	4.3
	Primary school	19	6.3
	Middle school	41	13.7
	High school	49	16.3
	Higher secondary	40	13.3
	Graduate	88	29.3
	Post graduate	50	16.7
Mother’s education	Illiterate	15	5.0
	Primary school	17	5.7
	Middle school	56	18.7
	High school	59	19.7
	Higher secondary	39	13.0
	Graduate	66	22.0
	Post graduate	48	16.0
Father’s occupation	Unemployed	1	0.3
	Elementary	38	12.7
	Plant and machine operators and assembler	10	3.3
	Craft and related trade workers	8	2.7
	Skilled agricultural and fishery workers	15	5.0
	Skilled workers and shop and market sales workers	96	32.0
	Clerks	13	4.3
	Technicians and associate professionals	70	23.3
	Professionals	39	13.0
	Legislators, senior officials and managers	10	3.3
Mother’s occupation	Unemployed	244	81.3
	Elementary	5	1.7
	Skilled workers and shop and market sales workers	8	2.7
	Clerks	1	0.3
	Technicians and associate professionals	19	6.3
	Professionals	19	6.3
	Legislators, senior officials and managers	4	1.3
Socioeconomic status	Upper	41	13.7
	Upper middle	52	17.3
	Lower middle	104	34.7
	Upper lower	103	34.3
Average time spent by parents with their child	<3 hours	10	3.3
	3–6 hours	52	17.3
	6–12 hours	52	17.3
	>12 hours	186	62.0
In absence of parents, child is attended by	Housemaid	8	2.7
	Other family members	292	97.3

The prevalence of excessive screen time was 57.7% (> 1 hour) in the study, and among them, the prevalence of excessive screen time was 39.3%, 13%, and 5.3% in children with screen time of one to three hours, three to five hours, and more than five hours, respectively. About 55% of children used smartphones, whereas 33.3% of children used both smartphones as well as television. The majority of children belonged to those over the age of one at the time of first exposure (73.7%), while 26.3% of children were exposed to screens before their first birthday. The majority of children used the device for entertainment purposes alone (65%), whereas 5.7% of children used the device for learning purposes. The child himself/herself was the deciding authority of the content to watch in 58% of cases, and the child was able to set the program partly or completely in 52.4% of cases. Safety of devices was ensured in 77.6% of cases. The preferred mode of entertainment was outdoor activities and friends in most cases (Table 2).

**Table 2:** Distribution according to prevalence and pattern of screentime

Screentime		Frequency (n=300)	Percentage
Average screen time	<1 hour	127	42.3
	1–3 hours	118	39.3
	3–5 hours	39	13.0
	>5 hours	16	5.3
Type of device	Smart phone	165	55.0
	TV	26	8.7
	Tablet	4	1.3
	Computer	2	0.7
	Smart phone, TV	100	33.3
	Tablet, TV	2	0.7
	Smart phone, TV, computer	1	0.3
Age at first exposure (months)	<3	8	2.7
	3–6	26	8.7
	6–12	45	15
	>12	221	73.7
Content	Cartoon/entertainment video	195	65.0
	Cartoon/entertainment video, learning videos	68	22.7
	Cartoon/entertainment video, video games	9	3.0
	Cartoon/entertainment video, violent video, video games	1	0.3
	Cartoon/entertainment video, learning videos, video games	1	0.3
	Cartoon/entertainment video, learning videos, violent content	1	0.3
	Learning videos	17	5.7
	Learning videos, video games	1	0.3
	Video games	4	1.3
	Violent or scary content	3	1.0
Deciding authority of content watched	Child	174	58.0
	Father	29	9.7
	Mother	93	31.0
	Mother/father	3	1.0
	Maid	1	0.3
Child set program	No	95	31.7
	Partly	62	20.7
	Yes	143	47.7
Safety of device ensured	Device locked	202	67.3
	Content locked	3	1.0
	Both	28	9.3
	None	67	22.3
Preferred entertainment if choice given	Devices	32	10.7
	Devices, family members	1	0.3
	Devices, toys	4	1.3
	Family members	59	19.7
	Friends	69	23.0
	Outdoor activities	86	28.7
	Toys	49	16.3

Table 3 represents the association of screen time with various behavioral, feeding, and sleep problems. If the device was not given to the child, restlessness was seen in 51.7% of children. Feeding and sleep problems were reported in 21.7% and 15.6% of cases, respectively.

**Table 3:** Distribution according to restlessness, feeding, and sleeping problems

Behavioral problems		Frequency (n=300)	Percentage
Restlessness if device not given	No	123	41.0
	Yes	155	51.7
	Cannot say	22	7.3
Feeding problems	No	219	73.0
	Yes	65	21.7
	Maybe	16	5.3
Change in sleeping pattern	No	253	83.3
	Yes, decreased	31	10.3
	Yes, increased	16	5.3

The mean PPSC score in the enrolled children was 6.7±3.3, and the risk of behavioral problems as per PPSC score (≥9 as positive) was present in 95 (31.7%) cases.

The risk of behavioral problems was significantly associated with higher screen time (p<0.05). Out of 16 children engaged in screen time for more than five hours per day, the risk of behavioral problems was present in the majority, which is 62.5% of children. The risk of behavioral problems was present in 56.4% of children with screen time of 3 to 5 hours, 37.3% of children with screen time of one to three hours, and 15% of children with screen time of less than 1 hour (Table 4).

**Table 4:** Association of risk of behavioral problems with average screen time

Average screen time	Risk of Behavioral problems absent		Risk of Behavioral problems present		Mean PPSC score
	Frequency	Percentage	Frequency	Percentage	
<1 hour	108	85.0	19	15.0	4.8±2.34
1–3 hours	74	62.7	44	37.3	6.93±3.57
3–5 hours	17	43.6	22	56.4	9.67±4.14
>5 hours	6	37.5	10	62.5	11.43±5.29
P-value	0.001				

As seen in Table 5, the risk of behavioral problems was significantly associated with less average time spent by parents with their child (p<0.05), i.e., the risk of behavioral problems was present in 50% and 40% of children whose parents spend time ranging from three to six hours and less than three hours, respectively, as compared to children of parents spending more time with children (p<0.05).

**Table 5:** Association of risk of behavioral problems in preschool children with average time spent by parents with their child

Average time given by parents to child	Risk of behavior problem			
	Absent (n=205)		Present (n=95)	
	n	%	n	%
<3 hours (n=10)	6	60.0	4	40.0
3–6 hours (n=52)	26	50.0	26	50.0
6–12 hours (n=52)	37	71.2	15	28.8
>12 hours (n=186)	136	73.1	50	26.9
χ^2^	10.557			
P-value	0.014			

The risk of behavioral problems in children using multiple devices in comparison to single-device users was statistically significant (Table 6). The risk of behavioral problems was present in 26.4% of children using single devices and 41.7% of children using multiple devices, and the observed difference was statistically significant (p<0.05).

**Table 6:** Association of risk of behavioral problems in preschool children with number of device used

Number of device	Risk of behavior problem			
	Absent (n=205)		Present (n=95)	
	n	%	n	%
Single device (n=197)	145	73.6	52	26.4
Multiple device (n=103)	60	58.3	43	41.7
χ^2^	7.36			
P-value	0.006			

Risk of behavioral problems was found in significantly higher proportions of children who preferred only devices (59.4%) and devices along with toys (50%) as the preferred source of entertainment when the choice was given, as compared to children preferring only toys (20.4%), outdoor activities (27.9%), and friends (29%) (p<0.05) (Table 7).

**Table 7:** Association of risk of behavioral problems in preschool children with preference of alternative source of entertainment

Preferences of entertainment if given choice	Risk of behavior problem			
	Absent (n=205)		Present (n=95)	
	n	%	n	%
Device (n=32)	13	40.6	19	59.4
Device, family member (n=1)	1	100.0	0	0.0
Devices, toys (n=4)	2	50.0	2	50.0
Family member (n=59)	39	66.1	20	33.9
Friends (n=69)	49	71.0	20	29.0
Outdoor activities (n=86)	62	72.1	24	27.9
Toys (n=49)	39	79.6	10	20.4
χ^2^	16.235			
P-value	0.013			

Table 8 shows that the risk of behavioral problems was present in significantly higher proportions of children becoming restless when the device was not given (42.6%) as compared to children not becoming restless (17.9%) (p<0.05).

**Table 8:** Association of risk of behavioral problems in preschool children with restlessness in child if device was not given

Child becomes restless if device not given	Risk of behavior problem			
	Absent (n=205)		Present (n=95)	
	n	%	n	%
No (n=123)	101	82.1	22	17.9
Yes (n=155)	89	57.4	66	42.6
Cannot say (n=22)	15	68.2	7	31.8
χ^2^	19.327			
P-value	0.001			

The present study found a significant association between the risk of behavioral problems with feeding problems and sleep changes, particularly decreased sleep. 67.7% of children whose parents had complained of decreased sleep time had a higher risk of behavior problems as opposed to those sleeping well. We also know that there is a well-established relationship between children who use screens more tend to sleep less and have feeding issues as well (p<0.05) (Table 9).

**Table 9:** Association of risk of behavioral problems in preschool children with feeding and sleeping problems

Problems		Risk of behavior problem				χ^2^	P-value
		Absent (n=205)		Present (n=95)			
		n	%	n	%		
Feeding	No (n=219)	161	73.5	58	26.5	11.09	0.004
	Yes (n=65)	37	56.9	28	43.1		
	Maybe (n=16)	7	43.8	9	56.2		
Sleep changes	No (n=253)	186	173.2	67	26.8	23.85	0.001
	Yes, decreased (n=31)	10	32.3	21	67.7		
	Yes, increased (n=16)	9	56.2	7	43.8		

No significant association of risk of behavior problems was seen with parents' education and occupation, socioeconomic status of family, age at first exposure to the screen, and safety of the device.

Finally, multivariate analysis is done in Table 10, which revealed higher odds of risk of behavioral problems in children with higher average screen time (1-3 hours OR: 1.536; 95% CI: 1.03-7.237; p=0.035, 3-5 hours OR: 2.022; 95% CI: 1.477-8.570; p=0.029, and >5 hours OR: 9.793; 95% CI: 2.065-46.447; p=0.004), children using multiple devices as compared to single devices (OR: 2.43; 95% CI: 1.194-4.95; p=0.014), and also in children whose parents spend less average time with their children (<3 hours OR: 1.311; 95% CI: 1.02-2.77; p=0.012).

**Table 10:** Risk factors associated with behavioral problems

Risk factors		OR	95% CI	P-value
Age (months)	18–24			
	25–36	0.99	0.32–3.06	0.98
	37–48	1.009	0.38–2.66	0.99
	49–60	1.41	0.64–3.16	0.39
Sex	Male	1.08	0.56–2.8	0.82
	Female	Reference		
Father’s education	More than primary	2.821	0.905–8.8	0.074
	Less than primary	Reference		
Mother’s education	More than primary	1.327	0.421–4.18	0.63
	Less than primary	Reference		
Father’s occupation	Employed	1.47	0.36–4.43.41	0.37
	Unemployed	Reference		
Mother’s education	Employed	0.740	0.312–1.75	0.49
	Unemployed	Reference		
Socioeconomic status	Class 1	0.950	0.283–3.28	0.933
	Class 2	0.940	0.353–2.50	0.901
	Class 3	1.60	0.677–3.79	0.284
	Class 4	Reference		
Average screen time	<1 hour	Reference		
	1–3 hours	1.536	1.03–7.237	0.035
	3–5 hours	2.022	1.477–8.570	0.029
	>5 hours	9.793	2.065–46.447	0.004
Type of device	Multiple device	2.430	1.194–4.95	0.014
	Single device	Reference		
Age at first exposure (months)	<3	0.303	0.036–2.54	0.271
	3–6	0.628	0.193–2.04	0.439
	6–12	0.845	0.342–2.09	0.715
	>12	Reference		
Content	Entertainment/ games	0.498	0.228–1.09	0.08
	Learning	Reference		
Deciding authority	Child	1.417	0.634–3.17	0.395
	Parents/others	Reference		
Child can set program	Yes/partly	0.774	0.322–1.86	0.57
	No	Reference		
Safety of device ensured	No	2.414	0.65–3.07	0.38
	Yes	Reference		
Average time spent by parents with their child	<3 hours	1.311	1.02–2.77	0.012
	3–6 hours	0.685	0.11–4.28	0.685
	6–12 hours	0.734	0.29–1.85	0.512
	>12 hours	Reference		

## Discussion

Children today live in a world where digital technology, such as mobile phones and interactive screen media, is ingrained in their lives. Prior studies have documented a higher prevalence of excessive screen time among children under five in high- and middle-income countries. Excessive screen time has detrimental effects on the health of children in the form of behavioral problems, sleep disorders, and emotional issues. It may affect growth and cognition in developing children. Some high-income countries, such as Canada, Italy, and Australia, have issued guidelines for limiting excessive usage of digital media for all age groups [12-14]. However, such guidelines are lacking in LMICs. We aimed to determine the prevalence and pattern of exposure to excessive screen time in children below five years and also to determine the prevalence of risk of behavioral problems in these children.

As children below two years of age are still developing language, sensorimotor, cognitive, and socioeconomic skills and require social interaction for average growth and development, the WHO and IAP recommend that children below two years should not be exposed to any type of screen and the screentime of one hour or more in children belonging to two to five years of age has been considered excessive screen time [4,6]. With the rise in new technologies, such as video gaming platforms, children and adolescents are increasingly exposed to virtual violence, where violence is not experienced physically but is experienced in realistic ways via several new technologies, and these are even more intense than realistic games [15].

In our study, the prevalence of excessive screen time was 57.7%, considering screen time of less than one hour as the norm. Various devices are available, such as mobile phones, televisions, tablets, etc., and most enrolled children use smartphones alone, followed by smartphones and televisions together. Kaur et al., in their review, documented the prevalence of excess screen time in the range of 10% to 93.7% in high-income countries and 21% to 98% in low and middle-income countries [7]. Jain et al., in their study, documented the prevalence of excessive screen time as 18%, considering >2 hours as excessive screen time [16]. The prevalence of excessive screen time in a study by Varadarajan et al. was 73.3% in children below two years of age and 73% in children over two years of age [17].

The mean age of children enrolled in the present study was 44.14±13.366 months, and the majority of children were between 49 and 60 months of age. We reported male predominance in the present study, with 56.7% of cases being males and most children belonging to the lower middle (34.7%) and upper lower socioeconomic class (34.3%). However, we observed no significant association of risk of behavioral problems with age, sex, or socioeconomic status of the child (p>0.05). Qu et al. reported a significant association of behavior problems in boys with excessive screen time [9]. However, no such association could be observed in the present study. Suleman et al. documented maternal education as a significant and independent predictor of behavioral problems in children (ß = 21.5; p<0.05) [18]. Chaturvedi et al., on the other hand, found a significant association between behavioral problems and low socioeconomic status. In contrast, time spent by the father and mother’s working status was associated with a positive impact on the behavior of the child (p<0.05) [19]. In the present study, none of the sociodemographic variables was independently associated with behavioral problems in children on multivariate analysis (p<0.05). 

Age at the time of exposure is an important factor determining behavioral problems in children. According to a study conducted in the United States, most children aged two years use a digital device every day, and electronic devices were introduced before their first birthday in 9 out of 10 children [20]. In our study, about three-fourths of children (73.7%) were exposed to screening after one year of age, whereas one-fourth of children were exposed before one year of age. In our study, about 65% of children used the screen to watch cartoons or entertainment videos, whereas one-third used the devices to watch entertainment and learning videos. As violent videos are associated with aggressive behavior in the child, it is essential for parents to ensure the safety of the device and decide the content to be watched. This may improve cognitive skills in children, as few learning videos have been shown to improve learning and cognitive skills [17]. In our study, though the safety of the device was ensured in 77.6% of cases as the device was password locked, the deciding authority regarding the content watched was the child in 58% of cases. According to a 2020 US survey, 39% of parents reported that television is always on (10%) or most of the time (29%). Children of these households are constantly watching television far more than other children of their age, with a risk of ill effects on development [21].

Screentime is associated with adverse effects on cognition, sleep, and behavior. The behavioral effect is observed mainly in children below five years of age, and among them, children below two years of age are the most susceptible. According to the American Academy of Pediatrics, screen time has potentially more negative effects as compared to positive effects in children below two years of age [4]. We used the PPSC score for assessing the risk of behavioral problems (≥9 as positive) in our study population, and the mean PPSC score in the enrolled children was 6.7±3.3. We reported a risk of behavioral problems in 31.7% of cases.

In the present study, restlessness, feeding issues, and sleeping issues were reported in 51.7%, 21.7%, and 15.7% of cases, respectively. We found a statistically significant association of the risk of behavioral problems with feeding issues, sleep disturbances, and restlessness when the device was not given (p<0.05). Kahn et al. reported a significant association between sleep, screen time, and behavioral problems in their study. Sleep duration of less than 9.88 hours was significantly associated with screen time and behavior problems. Sleep duration affected the externalizing behaviors in children but not internalizing behavior problems [22].

Our study documented a significant association between higher screen time and PPSC score, suggesting a higher risk of behavioral issues with higher screen time (p<0.05). Our study aimed to find out various risk factors associated with behavioral problems. Among them, excessive screen time, using multiple devices, and spending less time with parents were independently associated with a higher risk of behavioral problems among children two to five years of age. Parents' higher screen time may be associated with less time spent with children. Bahadur and Karaca found a significant association between higher maternal screen time and behavioral problems, particularly hyperactivity or inattention, in preschool children [23].

The findings of the present study were supported by the findings of Tamana et al., in which the authors documented significantly higher behavioral abnormalities and ADHD-like behavior in children spending screen time of two hours or more compared to children spending less than 30 minutes. According to this study, watching screens, particularly television, leads to the release of excessive dopamine, making it difficult to get rid of the electronic devices and thereby causing addiction [24]. Nathanson et al. reported that early age at the onset of media usage, greater hours of media usage, and poor quality content are independently associated with behavioral abnormalities in the form of poor executive functioning [25]. Chonchaiya and Pruksananonda documented that children with early exposure to TV and watching TV for more than two hours are six times more likely to develop linguistic difficulties [26].

Our study had certain limitations. First, the study may have had interviewer bias, recall bias, and reporting bias of parents, and we could not measure individual behavioral problems and other important variables such as temperament, parenting style, etc. Second, since the study was cross-sectional in nature, causal inferences and long-term adverse effects of the device's use could not be addressed.

## Conclusions

Digital media and digital devices are an indispensable part of our lives, and children in the digital native environment are exposed to a wide variety of screen-based devices early in their lives. Development is a continuous and ongoing process during the early years of life, and these years are the foundation of a child's behavior. The present study found that almost all children below five years are exposed to digital devices, with the majority of them exceeding the recommended screen time, and they mostly use it for entertainment purposes. During this period, exposure of children to screens is significantly associated with the risk of behavioral problems. It was observed that excessive screen time, less time spent by parents with their children, and use of multiple devices are significant predictors of increasing risk of behavioral problems in preschool children. A community-based prospective study is recommended to assess and observe the short-term and long-term effects of excessive screen time and early exposure to the screen.

Time spent by children with screens and the content they watch must be according to their developmental stage as recommended by various guidelines. Strict supervision by parents on screen time and the content being watched must be ensured by parents. Increased parental time and engagement in other activities may help overcome behavioral issues in children and prevent the long-term morbidities associated with screen time. It was seen that if children were given choices for entertainment, almost all children preferred outdoor activities, toys, or spending time with friends and family members, but the socio-demography of society has made them indulge in the digital world. Drafting government policy regarding screen time and public awareness regarding the same by the combined work of the government and healthcare workers can improve the situation in our country.
